# Association of α-Adducin and G-Protein β3 Genetic Polymorphisms with Hypertension: A Meta-Analysis of Chinese Populations

**DOI:** 10.1371/journal.pone.0017052

**Published:** 2011-02-25

**Authors:** Wenquan Niu, Yue Qi

**Affiliations:** 1 State Key Laboratory of Medical Genomics, Shanghai Key Laboratory of Vascular Biology and Department of Hypertension, Ruijin Hospital, Shanghai Jiao Tong University School of Medicine, Shanghai, China; 2 Laboratory of Vascular Biology, Institute of Health Sciences, Shanghai Institutes for Biological Sciences, Chinese Academy of Sciences, Shanghai, China; 3 Shanghai Institute of Hypertension, Shanghai, China; 4 Department of Epidemiology, Capital Medical University Affiliated Beijing Anzhen Hospital, Beijing Institute of Heart, Lung and Blood Vessel Diseases, Anzhenli, Beijing, China; University of California, San Francisco, United States of America

## Abstract

**Background:**

Mounting evidence has suggested that α-adducin and G-protein β3 (GNB3) genes are logical candidates for salt-sensitive hypertension. Some, but not all, studies have reported that α-adducin G460T and GNB3 C825T polymorphisms may influence the risk of the disease. To comprehensively address this issue, we performed a meta-analysis to evaluate the influence of these two polymorphisms on hypertension and potential biases in Chinese.

**Methods:**

Data were analyzed using Stata (v11.0) and random-effects model was applied irrespective of between-studies heterogeneity, which was evaluated via subgroup and meta-regression analyses. Study quality was assessed in duplicate. Publication bias was weighed using Egger's test and funnel plot.

**Results:**

36 study populations totaling 9042 hypertensive patients and 8399 controls were finally identified. Overall, in allelic/genotypic/dominant/recessive models, no significant association was identified for both G460T and C825T polymorphisms (P>0.05) and there was possible heterogeneity (*I* ^2^>25%). Subgroup analyses by study design indicated that the magnitude of association in population-based studies was marginally significantly strengthened for α-adducin G460T allelic model (OR = 1.12; 95% CI: 1:00–1.25; P = 0.043). Moreover, subgroup analyses by geographic distribution indicated comparison of 825T with 825C yielded a marginally significant increased risk in southern Chinese only (OR = 1.48; 95% CI: 1.01–2.16; P = 0.045). Further meta-regression analyses showed that geographic regions were a significant source of between-study heterogeneity for both polymorphisms. There was a possibility of publication bias for G460T, but not for C825T.

**Conclusions:**

Our overall results suggest null association of α-adducin G460T and GNB3 C825T polymorphisms with hypertension in Chinese but indicate local marginal significance of C825T, as a putative salt-sensitive switch, in southern Chinese.

## Introduction

A strong genetic underpinning presents in hypertension: premature onset of hypertension among first-degree relatives yielded a remarkable high risk of 3.8 times to develop hypertension [1]. However, the genetic profiles contributing to the huge amount of sporadic hypertension currently remain unclear. Evaluation of hypertension-susceptibility genes involving in a specific physiological or cellular function has therefore aroused much renewed interest.

Adducin exerts modulatory effects on the actin cytoskeleton dynamics and clathrin-dependent endocytosis [2]. The α-adducin gene (4p16.3) point mutation could affect renal sodium transport and explain a large proportion of blood pressure (BP) variation [3]–[5], suggesting the involvement of mutated adducin variants in sodium-dependent hypertension.

G-protein β3 (GNB3) subunit plays a vital role in intracellular signal transduction [6]. Concretely, the hetero-trimeric G-protein, via interacting with G-protein coupled receptors, can be dissociated into Gα-GTP and Gβγ complexes, resulting in a variety of cellular functions [7]. Additionally, mounting data have emerged from candidate gene approach favoring the susceptibility of GNB3 gene (12p13) to salt-sensitive hypertension [8]–[14].

As the genomic sequences of α-adducin and GNB3 genes are highly polymorphic, it is of added interest to identify which polymorphism(s) in these genes might have functional potentials of affecting their bioavailability. Two exonic polymorphisms G460T in α-adducin gene and C825T in GNB3 gene have become a focus of researches and are suggestively associated with hypertension, although this claim is controversial. Generally, inadequate inclusion of hypertensive patients and controls as well as genetic background noise might account for this controversy.

To generate more information, we restricted our research to Chinese populations and conducted a comprehensive meta-analysis to ascertain whether there was indeed an association of G460T and C825T polymorphisms with hypertension and whether study design and geographic distribution of study populations were potential biases and sources of heterogeneity between studies.

## Methods

### Literature Search

We collected information via two international searching engines, *viz*. PubMed and Excerpta Medica Database (EMBASE), as well as two Chinese searching engines, *viz*. Wanfang database (http://www.wanfangdata.com.cn) and China Biological Medicine (CBM) (http://cbm.imicams.ac.cn) with the last update as of September 20, 2010. As a prerequisite, only those published in English and Chinese languages and performed in Chinese populations were identified. In addition, reference lists of all retrieved publications were checked for the missing information. If two or more studies shared the same patients or control subjects, the one with large sample size was selected. If more than one geographical or ethnic populations were included in one report, each population was considered separately.

### Inclusion/Exclusion Criteria

Studies that we identified satisfied the following criteria: (i) evaluation of the α-adducin gene G460T or GNB3 gene C825T polymorphisms in association with hypertension; (ii) case-control study using either a hospital-based or a population-based design; (iii) sufficient information on G460T and C825T genotype counts between hypertensive patients and controls for estimating odds ratio (OR) and its corresponding 95% confidence interval (CI). Hypertension was defined as systolic BP equal to or above 140 mmHg or diastolic BP equal to or above 90 mmHg or treatment with antihypertensive medication. Studies evaluating secondary hypertension or other types of monogenic hypertension were excluded.

### Extracted Information

Two authors (W. Niu and Y. Qi) independently drew the following information from all qualified studies: first author's last name, publication date, population ethnicity, study design, baseline characteristics of the study population, and the genotype distribution in patients and controls. Any encountered discrepancies were adjudicated by a discussion until a consensus was reached. For consistency, continuous variables e.g. age expressed as mean ± standard error (SE) were converted to mean ± standard deviation (SD).

### Statistical Analysis

Pooled allelic/genotypic association of α-adducin gene G460T and GNB3 gene C825T polymorphisms with hypertension was performed using the Stata/SE 11.0 for Windows. In this meta-analysis, we implemented the random-effects model using the method of DerSimonian & Laird, instead of fixed-effects model, to bring the individual effect-size estimates together, and the estimate of heterogeneity was taken from the Mantel-Haenszel model [15], [16]. Satisfaction of the genotype distributions with Hardy-Weinberg proportions was performed using the SAS 9.1.3 for Windows in both cases and controls of all studies. This law is destroyed if the χ^2^-based probability is less than 0.05.

Heterogeneity was assessed by the *I* ^2^ statistic, which was documented for the percentage of the observed between-study variability due to heterogeneity rather than chance with the ranges of 0 to 100% *[I* ^2^ = 0–25%, no heterogeneity; *I* ^2^ = 25–50%, moderate heterogeneity; *I* ^2^ = 50–75%, large heterogeneity; *I* ^2^ = 75–100%, extreme heterogeneity] [17].

In addition, to look at more narrowly drawn subsets of the studies such as different study designs and areas, separate analyses were undertaken in a sensitivity way. Furthermore, to estimate the extent to which one or more covariates explain heterogeneity, meta-regression, as an extension to random-effects meta-analysis, was employed. The meta-regression model relates the treatment effect to the study-level covariates, assuming a normal distribution for the residual errors with both a within-study and an additive between-studies component of variance.

Finally, publication bias was assessed using the Egger regression asymmetry test. The Egger's test detects funnel plot asymmetry by determining whether the intercept deviates significantly from zero in a regression of the standardized effect estimates against their precision.

## Results

### Studies and Populations

As for α-adducin gene G460T, we identified 23 eligible reports published both in Chinese (n = 20) and English (n = 4), which met our inclusion/exclusion criteria, whereas due to the shared study groups, we removed five of them with the fewer sample sizes. Finally, there were 18 qualified studies (18 populations) totaling 4676 hypertension patients and 4482 controls. Basic characteristics of the study populations are presented in Table S1. Genotype distributions of G460T satisfied the Hardy-Weinberg equilibrium in both cases and controls for all qualified studies (P>0.05).

Regarding GNB3 gene C825T, a total of 29 reports were identified, and 11 of them were excluded for the following reasons: five papers with shared study subjects, four review articles, one with only hypertensive patients, and one conducted in Japanese subjects. Of the remaining 18 qualified reports (20 populations), 3 were published in English and 15 in Chinese. Basic characteristics of the study populations are presented in Table S2. Because two populations with control groups deviating from Hardy-Weinberg law was removed, there was finally 18 populations involving 4366 hypertensive patients and 3917 controls.

**Figure 1:**
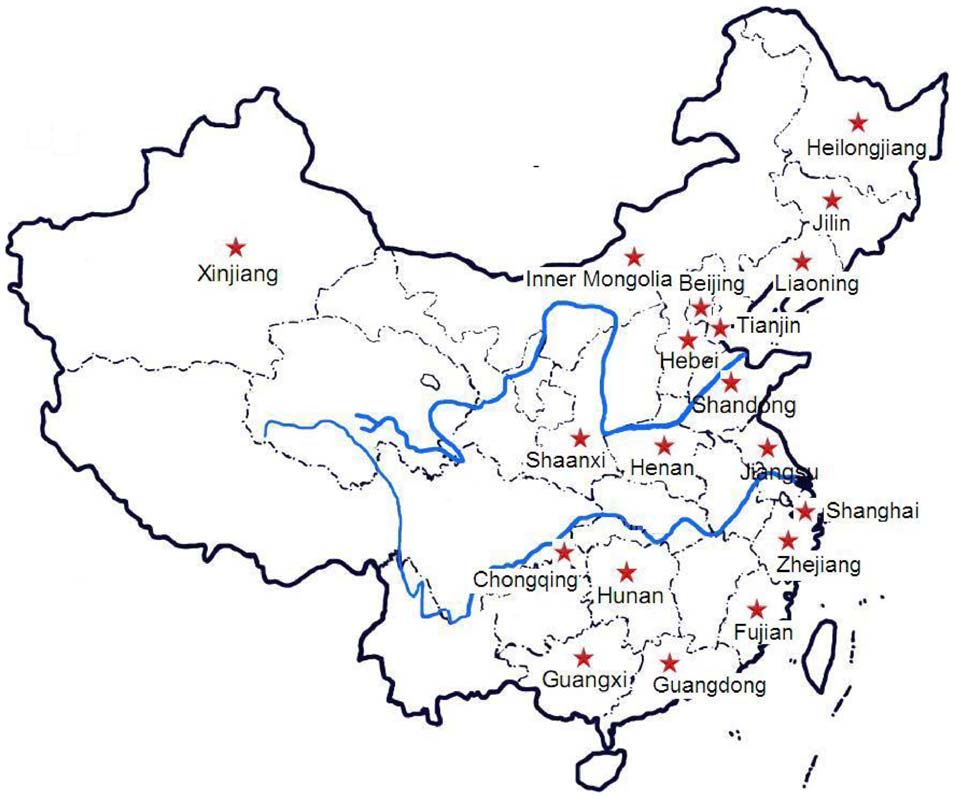
The geographical distributions (as marked by pentagram) of all eligible studies in China map.

Distribution of the population sources for all studies was shown in Figure 1. The participants were recruited from 19 provinces and municipalities directly under the central government.

### Pooled Analyses

As for α-adducin gene G460T, in allelic model, comparison of 460T with 460G allele generated a nonsignificant 5% increased hypertension risk (P = 0.34) (Table 1). No significance was observed in genotypic models for comparisons of 460GT (OR = 1.00; P = 0.945) and 460TT (OR = 1.09; P = 0.94) genotypes with 460GG genotype, respectively, as well as in dominant (OR = 1.04; P = 0.496) and recessive (OR = 1.05; P = 0.528) models. The *I* ^2^ statistic indicated the large between-study heterogeneity for all comparisons except for that of 460GT with 460GG (*I* ^2^ = 2.7%).

**Table 1:** Meta-analysis of the association between α-adducin gene G460T polymorphism and hypertension risk.

Comparisons	Pooled OR (95% CI)	Z (P)	*I* ^2^ (%)
460T vs. 460G	1.05 (0.95, 1.16)	0.95 (0.340)	58.6
460GT vs. 460GG	1.00 (0.91, 1.11)	0.07 (0.945)	2.7
460TT vs. 460GG	1.09 (0.90, 1.32)	0.84 (0.402)	56.8
460GT+TT vs. 460GG	1.04 (0.92, 1.18)	0.68 (0.496)	34.7
460TT vs. 460GT+460GG	1.05 (0.90, 1.23)	0.63 (0.528)	53.9

Abbreviations: OR, odds ratio; CI, confidence interval.

With regard to GNB3 gene C825T, in allelic model, comparison of 825T with 825C allele yielded a nonsignificant 2% increased risk (P = 0.56) (Table 2). Similarly no significance was observed in genotypic models for comparisons of 825CT (OR = 1.05; P = 0.4) and 825TT (OR = 1.01; P = 0.858) genotypes with 825CC genotype, respectively, as well as in dominant (OR = 1.05; P = 0.417) and recessive (OR = 0.99; P = 0.908) models. No between-study heterogeneity was identified using *I* ^2^ statistic except for the dominant model (*I* ^2^ = 31.6%).

**Table 2:** Meta-analysis of the association between GNB3 gene C825T polymorphism and hypertension risk.

Comparisons	Pooled OR (95% CI)	Z (P)	*I* ^2^ (%)
825T vs. 825C	1.02 (0.95, 1.10)	0.58 (0.560)	24.7
825CT vs. 825CC	1.05 (0.93, 1.19)	0.84 (0.400)	18.8
825TT vs. 825CC	1.01 (0.89, 1.15)	0.18 (0.858)	5.2
825CT+TT vs. 825CC	1.05 (0.93, 1.19)	0.81 (0.417)	31.6
825TT vs. 825CT+825CC	0.99 (0.90, 1.10)	0.12 (0.908)	0.0

*Abbreviations*: OR, odds ratio; CI, confidence interval.

Moreover, since most of studies were conducted in Han Chinese, after restricting analyses to Han populations, there was no material changes in ORs for both polymorphisms under study (data not shown).

### Subgroup Analyses

**Figure 2:**
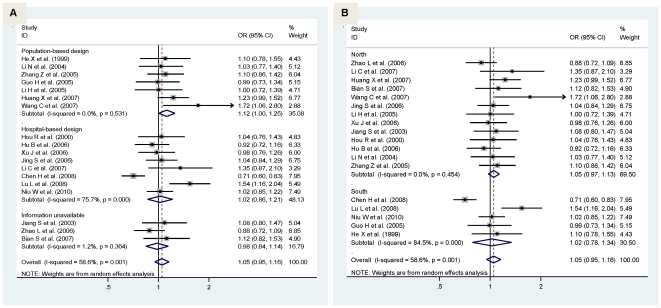
Pooled random-effects-based odds ratio of developing hypertension for α-adducin gene 460T allele versus 460G allele by study design (pane A) and area (pane B).

**Figure 3:**
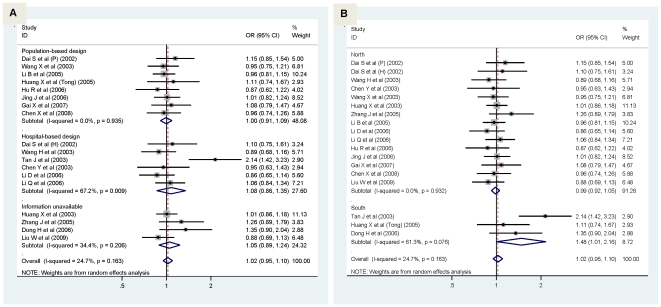
Pooled random-effects-based odds ratio of developing hypertension for GNB3 gene 825T allele versus 825C allele by study design (pane A) and area (pane B).

Considering the fact that study design and the geographic difference might bias the overall association, we conducted separate analyses according to these factors. In view of study design, the magnitude of association in population-based studies was marginally significantly strengthened for α-adducin gene G460T allelic model (OR = 1.12; 95% CI: 1:00–1.25; P = 0.043) (Figure 2-A), but weakened for GNB3 gene C825T allelic model (Figure 3-A). Nearly no changes in ORs was observed in hospital-based studies for both polymorphisms.

China has a vast territory, and geographic differences cause various characters as a result of historical and cultural impact. We thus demarcate China into north and south by the Qinling Mountains of China's Huaihe River. In allelic model, as for G460T, there was no material difference between northern and southern Chinese (Figure 2-B), whereas for C825T, remarkable changes were observed with comparison of 825T with 825C yielding a marginally significant increased risk in southern Chinese only (OR = 1.48; 95% CI: 1.01–2.16; P = 0.045) (Figure 3-B).

In case of genotypic, dominant and recessive models, there were no material changes in prediction of these two polymorphisms for hypertension according to study design and the geographic difference (data not shown).

### Meta-Regression Analyses

To evaluate the extent to which different variables explained heterogeneity among the individual ORs, we performed two models, *viz*. model 1 regressing study design, geographic region, and the differences of age, gender and body mass index (BMI) between cases and controls; model 2 regressing study design, geographic region, and the average of age, gender and BMI of both groups (Table 3).

**Table 3:** Meta-regression analysis of α-adducin gene G460T and GNB3 gene C825T polymorphisms with covariates of interest.

Variables	α-adducin gene G460T				GNB3 gene C825T			
	Coef.	95% CI	t	P	Coef.	95% CI	t	P
**Model 1**								
Study design	0.23	(−0.10, 0.56)	2.24	0.111	−0.14	(−0.34, 0.06)	−2.01	0.115
Area	0.21	(−0.01, 0.43)	3.02	0.057	0.21	(−0.03, 0.45)	2.41	0.074
Diff-age	−0.16	(−0.44, 0.11)	−1.90	0.154	−0.04	(−0.10, 0.02)	−1.74	0.156
Diff-gender	−1.79	(−6.49, 2.92)	−1.21	0.314	−0.003	(−0.03, 0.03)	−0.27	0.797
Diff-BMI	−0.16	(−0.37, 0.04)	−2.56	0.083	−0.001	(−0.18, 0.18)	−0.03	0.980
**Model 2**								
Study design	−0.11	(−0.81, 0.60)	−0.49	0.659	0.02	(−0.21, 0.24)	0.19	0.859
Area	0.19	(−0.28, 0.65)	1.28	0.290	0.82	(0.04, 1.59)	2.94	0.043
Ave-age	−0.04	(−0.13, 0.05)	−1.29	0.287	−0.01	(−0.03, 0.18)	−0.70	0.522
Ave-gender	0.34	(−2.53, 3.20)	0.37	0.734	−0.001	(−0.02 0.02)	−0.11	0.919
Ave-BMI	0.06	(−0.11, 0.23)	1.16	0.330	−0.02	(−0.07, 0.02)	−1.28	0.270

*Abbreviations*: Coef., coefficient; CI, confidence interval; BMI, body mass index. Diff-age (-gender and -BMI) means the age (percent of male and BMI) differences between cases and controls (Model 1). Ave-age (-gender and -BMI) means the average age (percent of male and BMI) of both groups (Model 2).

As for G460T, geographic region (Coef = 0.21; P = 0.057) and BMI (Coef = −0.16; P = 0.083) were a marginally significant source of between-study heterogeneity in model 1 and no heterogeneity was identified in model 2. In contrast, for C825T, meta-regression suggested that geographic region could increase the risk of hypertension in southern Chinese compared with northern ones in both model 1 (Coef = 0.21; P = 0.074) and model 2 (Coef = 0.82; P = 0.043).

### Publication Bias

**Figure 4:**
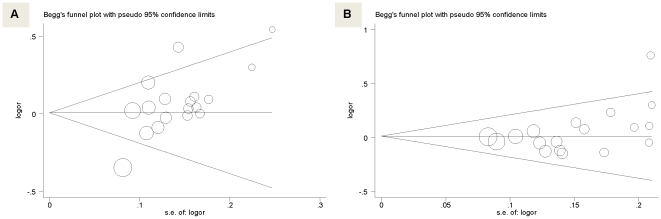
Funnel plots for studies investigating the effect of α-adducin gene G460T (pane A) and GNB3 gene C825T (pane B) polymorphisms on the risk of hypertension. Vertical axis represents the log of OR; horizontal axis represents the SE of log(OR). Funnel plots are drawn with 95% confidence limits. OR, odds ratio; SE, standard error. The graphic symbols represents the data in the plot be sized proportional to the inverse variance.

As reflected by the Egger's test and funnel plot (Figure 4), there was a possibility of publication bias for α-adducin gene G460T polymorphism (P = 0.004) and no publication bias for GNB3 gene C825T polymorphism (P = 0.069).

## Discussion

Via a comprehensive meta-analysis with 36 study populations totaling 17441 subjects, we assessed association of α-adducin gene G460T and GNB3 gene C825T polymorphisms with hypertension in Chinese and we failed to detect the overall positive signals for these two polymorphisms. Compared with the large volumes of evidence implicating their biological involvement in BP regulation, some, but not all, studies have positively ascertained the relationship between these polymorphisms and human hypertension [18]–[21]. Besides a possible role of ethnic differences in genetic profiles, other factors such as people living environment and dietary habits contribute as well [22].

More recently, a larger meta-analysis involving 22 worldwide studies by Liu et al failed to observe any signs of significance for association of α-adducin gene G460T polymorphism with hypertension [23], which was in agreement with the presents results of overall allelic/genotypic models in Chinese, whereas our subgroup analyses by study design yielded significant association signals in population-based studies only. Generally, in hospital-based studies, poor comparability between cases and controls might exert a confounding effect on the true association in light of a regional specialty for the disease under study and the differential hospitalization rates between cases and controls [24]. Because in this meta-analysis, most studies have recruited subjects from only one hospital, there may be a narrow socioeconomic profile for both patients and controls. Contrastingly controls drawn from the general population might be representative of the true population of those without the disease [25]. It is thus reasonably believed that results from population-based studies might hold the water, leading us to speculate that α-adducin gene G460T polymorphism might be a logical candidate for hypertension susceptibility in Chinese.

It is generally believed that except a climatically different environment between northern and southern China, dietary habits especially salt consumption make a great difference [26]. Indeed on the grounds of a previous observation in 2 northern Chinese sites and 1 in southern China, the average urinary sodium excretion in northern Chinese (271 mmol/d) was nearly twice that of southern adults (139 mmol/d), leading to an average 7.4 mmHg systolic and 6.9 mmHg diastolic BP higher in the northern subjects than southern ones [27]. Although experimental studies indicated the α-adducin gene locus was closely relevant to renal sodium transport [2], [28], [29], we failed to provide evidence for the geographic predisposition of α-adducin gene G460T to hypertension. This might be due to a significant possibility of publication bias and heterogeneity between studies. We agree that confirmation in large Chinese populations is critical.

In addition, a recent report by Kelly et al in Han Chinese suggested a role for the α-adducin gene and GNB3 gene in blood pressure salt sensitivity [30], which is in line with our results in GNB3 gene C825T polymorphism for both subgroup and meta-regression analyses. Since in only southern Chinese, we found marginally significant association of 825T with increased hypertension risk, whereas direction of this association was totally reversed and the magnitude was greatly alleviated in northern Chinese. In view of this difference, we thus reasoned that GNB3 gene C825T polymorphism might serve as a putative molecular switch for salt sensitivity, and under stimulation of high-salt diet, the functional effect of GNB3 gene harboring 825T allele is weakened or inhibited. It is also expected that multiple genetic and/or physiological safeguards that curb the adverse effect of GNB3 gene have developed to maintain blood pressure within a normal range of acceptable levels. Considering the relative small sample sizes of Southern Chinese in this study, our results should be considered preliminary, and cannot be directly extrapolated to contribution of this polymorphism to hypertensive patients.

Despite the clear strength of our study including large sample sizes, some limitations merit serious consideration. First, all included studies had the cross-sectional design, which precludes further comments on cause-effect relationship [31]. Second, for hypertension association studies, most studies have recruited subjects aged ≥50 years, for whom environmental factors are likely to contribute more prominently than the genetic component to the development of hypertension, suggesting that large association studies in a younger Chinese population of hypertensive subjects are of added interest. Third, due to the relative small number of some studies, we were unable to perform further subgroup analyses such as by gender and age groups. Further we cannot retrieve common information from all these original publications upon important confounding factors such as dietary salt intake in meta-regression models. Fourth, the single-locus-based nature of meta-analysis precluded the possibility of gene-gene and gene-environment interactions, as well as haplotype-based effects in this study, suggesting that additional studies assessing these aspects will be necessary. Last but not least, in this study, we only focused on α-adducin gene G460T and GNB3 gene C825T polymorphisms, and did not evaluate other genes or polymorphisms responsible for sodium-dependent hypertension. It is possible that the potential role of G460T or C825T polymorphism is diluted or masked by other gene-gene or gene-environment interactions. Thus, the jury must refrain from drawing a conclusion until a large, well-performed Chinese study confirms or refuses our results.

Taken together, our overall associations suggested null association of α-adducin G460T and GNB3 C825T polymorphisms with hypertension in Chinese, whereas we locally indentified marginal significance for C825T, as a putative salt-sensitive switch, in southern Chinese. Although further analyses are warranted to investigate α-adducin gene and GNB3 gene adjacent markers in a wider context, future studies on GNB3 and hypertension should concentrate on gene-environment interactions, particularly on the influence of 825T variant on hypertension or blood pressure in populations with different salt intake habits.

## Supporting Information

Table S1 The baseline characteristics of all qualified studies for α-adducin gene G460T polymorphism in this meta-analysis.
(DOC)

Table S2 The baseline characteristics of all qualified studies for GNB3 gene C825T polymorphism in this meta-analysis.
(DOC)
