# Supplementary material for: Association of α-Adducin and G-Protein β3 Genetic Polymorphisms with Hypertension: A Meta-Analysis of Chinese Populations
Source: PLoS One. 2011 Feb 25;6(2):e17052. doi: 10.1371/journal.pone.0017052 (PMC3045422; doi:10.1371/journal.pone.0017052)
Supplement: Table S1 — The baseline characteristics of all qualified studies for α-adducin gene G460T polymorphism in this meta-analysis. (DOC) [file pone.0017052.s001.doc]

***Supplementary Table 1.*** The baseline characteristics of all qualified studies for α-adducin gene G460T polymorphism in this meta-analysis

| **Study** | **Region** | **Nationality** | **Study Design** | **Status** | **Age (years)** | **M/F** | **BMI (kg/m^2^)** | **SBP (mmHg)** | **DBP (mmHg)** |
| --- | --- | --- | --- | --- | --- | --- | --- | --- | --- |
| Li C et al.  2007 | Tianjin | Han | hospital-based | Cases | —^*^ | — | — | — | — |
|  |  |  |  | Controls | — | — | — | — | — |
| Huang X et al. 2007 | Shandong | Han | population-based | Cases | 44±6 | 130/126 | 27±3 | 150±16 | 101±8 |
|  |  |  |  | Controls | 45±5 | 260/235 | 24±3 | 115±11 | 75±8 |
| Jing S et al. 2006 | Beijing | Han | hospital-based | Cases | 51.9±10.2 | 249/214 | — | 147.5±13.0 | 98.4±4.5 |
|  |  |  |  | Controls | 51.4±9.0 | 142/118 | — | 118.4±11.1 | 74.3±5.8 |
| Hu B et al. 2006 | Shanghai | Han | hospital-based | Cases | 56.5±11.5 | 275/121 | 25.6±3.6 | 157.3±17.4 | 96.6±12.1 |
|  |  |  |  | Controls | 56.2±6.5 | 146/68 | 23.9±3.1 | 119.5±9.2 | 78.6±6.2 |
| Guo H et al. 2005 | Zhejiang | She | population-based | Cases | — | — | — | — | — |
|  |  |  |  | Controls | — | — | — | — | — |
| Zhang Z et al. 2005 | Xinjiang | Kazakh | population-based | Cases | 48.8±10.9 | 135/143 | 24.9±3.5 | 165.7±22.5 | 105.6±13.5 |
|  |  |  |  | Controls | 48.1±10.2 | 87/133 | 24.3±3.1 | 118.1±13.5 | 77.2±7.9 |
| Hou R et al. 2000 | Shaanxi | Han | hospital-based | Cases | 47.6±7.8 | 97/86 | — | 151.7±13.5 | 96.4±12.1 |
|  |  |  |  | Controls | 46.6±7.4 | 68/61 | — | 114.3±10.0 | 74.4±9.2 |
| He X et al. 1999 | Shanghai | Han | population-based | Cases | 50.7±7.6 | 82/56 | 24.8±2.7 | 146±17 | 98±11 |
|  |  |  |  | Controls | 49.4±4.7 | 73/48 | 22.6±2.6 | 111±10 | 74±7 |
| Lu L et al. 2008 | Fujian | Han | hospital-based | Cases | 49.78±13.23 | 102/98 | 23.19±3.65 | 140±19.3 | 82.5±15.3 |
|  |  |  |  | Controls | 49.06±9.98 | 102/98 | 23.54±3.15 | 122±10.8 | 66.7±8.6 |
| Niu W et al. 2010 | Shanghai | Han | hospital-based | Cases | 52.71±4.08 | 251/224 | 25.45±2.78 | 141.61±14.90 | 94.98±8.37 |
|  |  |  |  | Controls | 52.74±4.22 | 241/234 | 23.44±2.81 | 115.51±10.21 | 76.86±6.14 |
| Chen H et al. 2008 | Fujian | Han | hospital-based | Cases | 58.0±10.0 | 662/418 | — | — | — |
|  |  |  |  | Controls | 57.4±11.1 | 342/262 | — | — | — |
| Xu J et al. 2006 | Beijing | Han | hospital-based | Cases | 49.28±9.43 | 186/162 | — | — | — |
|  |  |  |  | Controls | 50.19±10.55 | 101/83 | — | — | — |
| Bian S et al. 2007 | Hebei | Han | — | Cases | 64.29±5.19 | 71/89 | — | — | — |
|  |  |  |  | Controls | 62.04±5.67 | 78/73 | — | — | — |
| Zhao L et al. 2006 | Shanghai | Han | — | Cases | 56.55±12.55 | 149/129 | — | — | — |
|  |  |  |  | Controls | 51.00±5.22 | 128/103 | — | — | — |
| Jiang S et al. 2003 | Jiangsu | Han | — | Cases | 56.9±11.0 | 105/84 | — | 138.0±16.9 | 80.0±10.3 |
|  |  |  |  | Controls | 49.8±9.6 | 49/98 | — | 116.0±10.6 | 68.0±7.4 |
| Wang C et al. 2007 | Inner Mongolia | Mongol | population-based | Cases | 52.72±14.31 | 55/45 | — | 150.58±22.01 | 97.71±13.85 |
|  |  |  |  | Controls | 56.64±9.69 | 30/20 | — | 118.45±13.87 | 74.12±9.37 |
| Li H et al.  2005 | Xinjiang | Kazakh | population-based | Cases | 48.9±6.99 | 74/127 | 26.36±4.67 | 173.43±26.92 | 107.58±16.28 |
|  |  |  |  | Controls | 47.5±5.94 | 44/68 | 24.79±5.07 | 117.05±10.80 | 73.89±7.20 |
| Li N et al.  2004 | Xinjiang | Kazakh | population-based | Cases | 48.34±9.67 | 91/144 | 26.01±4.59 | 171.86±26.51 | 106.37±16.39 |
|  |  |  |  | Controls | 48.24±8.61 | 51/81 | 24.58±4.87 | 116.70±10.92 | 73.67±7.29 |

***Abbreviations:*** M/F, males/females; BMI, body mass index; SBP, systolic blood pressure; DBP, diastolic blood pressure.

Data are expressed as mean ± standard deviation (SD). * information not available.

***References***

1. Li C, Dang Q, Mu H (2007) Association study between essential hypertension and a-adducin P460Trp mutation. Guoji Xin Xue Guan Bing Za Zhi 34: 472-474. [Article in Chinese]
2. Huang XH, Sun K, Song Y, Zhang HY, Yang Y et al. (2007) Association of alpha-adducin gene and G-protein beta3-subunit gene with essential hypertension in Chinese. Zhonghua Yi Xue Za Zhi 87: 1682-1684. [Article in Chinese]
3. Jing S, Sun NL, Wang HY, Ma Z (2006) Association of the alpha-adducin Gly460Trp polymorphism with essential and blood pressure – lowing response to valsartan hydrochlorothiazide. Zhongguo Lin Chuang Yao Li Xue Za Zhi 22: 112-115. [Article in Chinese]
4. Hu BC, Chu SL, Wang JG, Wang GL, Gao PJ, et al. (2006) Single nucleotide polymorphisms of three candidate genes in essential hypertension. Zhonghua Nei Ke Za Zhi 45: 281-284. [Article in Chinese]
5. Guo HF, Li Y, Wang GL, Lu YG, Zhou HF, et al. (2005) Association of peripheral and central blood pressure with the alpha-adducin Gly460Trp polymorphism in a Chinese population. Zhonghua Xin Xue Guan Bing Za Zhi 33: 608-612. [Article in Chinese]
6. Zhang ZX, Liu Y, Lin RY, Wang SZ, Wang XF, et al. (2005) Connection between ADD1 Gene Gly460Trp Polymorphism and Essential Hypertension from Xinjiang Kazakhs. Fudan Xue Bao (Yi Xue Ban) 32: 239-242. [Article in Chinese]
7. Hou R, Liu ZQ, Xue MZ, Wang YX, Ye T, et al. (2000) Is Gly460Trp variant of alpha-adducin associated with essential hypertension in the Hans of Chinese population? Zhonghua Yi Xue Yi Chuan Xue Zha Zhi 17: 413-416. [Article in Chinese]
8. He X, Chu SL. Jin L, Xiong MM, Wang GL, et al. (1999) The relationship between alpha-adducin gene polymorphism and essential hypertension. Gao Xue Ya Za Zhi 7: 308-311. [Article in Chinese]
9. Lu LH, Chen H, Yu L (2008) Association of alpha-adducin and angiotensin converting enzyme gene polymorphisms with salt-sensitive hypertension and early renal injury. Zhonghua Yi Xue Yi Chuan Xue Za Zhi 25: 132-135. [Article in Chinese]
10. Niu WQ, Zhang Y, Ji KD, Gao PJ, Zhu DL (2010) Lack of association between alpha-adducin G460W polymorphism and hypertension: evidence from a case-control study and a meta-analysis. J Hum Hypertens 24: 467-474.
11. Chen H, Lin HZ, Chen Y, Lou JW, Wu XY, et al. (2008) Relationship of gene polymorphisms of angiotensin converting enzyme, aldosterone synthase and alpha-adducin with subclinical renal lesion. Zhonghua Lao Nian Yi Xue Za Zhi 27: 653-656. [Article in Chinese]
12. Xu J, Hua Q, Li DB, Liu RK, Yang Z, Chen HL, Guo JC. Relationship between alpha-adducin gene Gly460Trp polymorphism and the nursing intervention of hydrochlorothiazide. *Xin Xue Guan Bing Za Zhi* 2006; 25:158-160. [Article in Chinese]
13. Bian SH, Geng Q, Yu MY, Zhang Y, Xie YT. Relationship between alpha-adducin gene polymorphism and essential hypertension. *Lin Chuang Xin Xue Guan Bing Za Zhi* 2007; 23:897-899. [Article in Chinese]
14. Zhao LQ, Gao PJ, Zhu DL. Relationship between combination effect of ACE and adducin genes and essential hypertension. *Zhongguo Xin Xue Guan Za Zhi* 2006; 11:16-19. [Article in Chinese]
15. Jiang SH, Li XL, Zhang HF, Wang ZZ, De L, Yuan L, Huang J. Polymorphism of alpha-adducin, angiotensin-converting enzyme (ACE) genes, environmental factors and essential hypertension. *Gao Xue Ya Za Zhi* 2003; 11:563-566. [Article in Chinese]
16. Wang C, Sun G, Yan XL, Ding YC. Study of alpha-adducin and endothelial nitric oxide synthase gene polymorphism in patients with essential hypertension in Mongolia population. *Lin Chuang Xin Xue Guan Bing Za Zhi* 2007; 23:525-527. [Article in Chinese]
17. Li HJ, Li NF, Zhou L, Yin XJ, Shi Y, Cao M, Li T. The relationship between the alpha-adducin gene variant and essential hypertension in Kazaks. *Xinjiang Yi Ke Da Xue Xue Bao* 2005; 28:822-824. [Article in Chinese]
18. Li NF, Li HJ, Zhou L, Nu EG, Ouyang WJ, Bu KL, *et al*. The erlationship between the alpha-adducin gene variant and essential hypertension in Kazakans of Xinjiang. *Ke Xue Ji Shu Yu Gong Cheng* 2004; 7:551-555. [Article in Chinese]
