# Supplementary material for: Association of α-Adducin and G-Protein β3 Genetic Polymorphisms with Hypertension: A Meta-Analysis of Chinese Populations
Source: PLoS One. 2011 Feb 25;6(2):e17052. doi: 10.1371/journal.pone.0017052 (PMC3045422; doi:10.1371/journal.pone.0017052)
Supplement: Table S2 — The baseline characteristics of all qualified studies for GNB3 gene C825T polymorphism in this meta-analysis. (DOC) [file pone.0017052.s002.doc]

***Supplementary Table 2.*** The baseline characteristics of all qualified studies for GNB3 gene C825T polymorphism in this meta-analysis

| **First author** | **Year** | **Nationality** | **Study design** | **Area** | **Status** | **Number** | **age, year** | **gender (M)** | **Blood pressure** | | **BMI, kg/m^2^** |
| --- | --- | --- | --- | --- | --- | --- | --- | --- | --- | --- | --- |
|  |  |  |  |  |  |  |  |  | **SBP, mmHg** | **DBP, mmHg** |  |
| Dai S et al. | 2002 | Han | population | Liaoning province | Cases | 133 |  | 51.13% | 153.48 (24.79) | 100.05 (10.63) |  |
|  |  |  |  |  | Controls | 257 |  | 33.46% | 118.70 (13.93) | 76.01 (7.56) |  |
|  |  | Han | hospital | Shenyang city, Liaoning province | Cases | 98 |  | 36.74% |  |  |  |
|  |  |  |  |  | Controls | 110 |  | 39.09% |  |  |  |
| Wang H et al. | 2003 | Han | hospital | Beijing city | Cases | 408 | 54.63 (12.39) | 52.7% |  |  |  |
|  |  |  |  |  | Controls | 140 | 43.52 (14.53) | 65% |  |  |  |
| Tan J et al. | 2003 | Han | hospital | Chongqing city | Cases | 112 | 59 (7) | 55.36% | 155.6 (17.1) | 88.4 (11.34) | 24.3 (3.6) |
|  |  |  |  |  | Controls | 112 | 61 (8) | 55.36% | 122.3 (10.9) | 74.9 (7.9) | 23.5 (3.1) |
| Chen Y et al. | 2003 | Han | hospital | Daqing city, Heilongjiang province | Cases | 90 |  |  |  |  |  |
|  |  |  |  |  | Controls | 99 |  |  |  |  |  |
| Wang X et al. | 2004 | Kazakh | population | Barkol county, Xinjiang province | Cases | 290 | 48.7 (11) | 48.28% | 165.4 (22.3) | 105.5 (13.4) | 24.9 (3.5) |
|  |  |  |  |  | Controls | 244 | 47.2 (10.3) | 39.75% | 118.1 (13.3) | 76.7 (7.9) | 24.4 (3) |
| Huang X et al. | 2003 | Han | hospital | Beijing city | Cases | 585 | 51.4 (7.9) | 55.56% | 149 (18) | 77 (7) | 26.0 (3.5) |
|  |  |  |  |  | Controls | 580 | 53.3 (6.7) | 55% | 119 (11) | 95 (11) | 24.2 (3.4) |
| Huang X et al. | 2005 | Tong | population | Huaihua city, Hunan province | Cases | 96 |  | 44.79% |  |  |  |
|  |  |  |  |  | Controls | 89 |  | 46.07% |  |  |  |
|  |  | Han* | population | Huaihua city, Hunan province | Cases | 76 |  | 52.63% |  |  |  |
|  |  |  |  |  | Controls | 151 |  | 47.68% |  |  |  |
| Zhang J et al. | 2005 | Han | NA | Zhengzhou city, Henan province | Cases | 110 | 58.3 (5.4) | 52.73% |  |  |  |
|  |  |  |  |  | Controls | 150 |  |  |  |  |  |
| Li B et al. | 2005 | Han | population | InterASIA (Beijing, Jilin, Shandong) | Cases | 501 | 53.6 (9.3) | 52.1% | 177.0 (28) | 104.3 (12.3) | 26.3 (3.85) |
|  |  |  |  |  | Controls | 503 | 53.7 (9.2) | 52.09% | 117.6 (11.6) | 75.1 (8.0) | 24.34 (3.58) |
| Li D et al. | 2006 | Han | hospital | Beijing city | Cases | 310 | 47.99 (9.9) | 51.61% |  |  |  |
|  |  |  |  |  | Controls | 151 | 47.14 (12.3) | 50.99% |  |  |  |
| Dong H et al. | 2006 | Han | NA | Shenzhen city, Guangdong province | Cases | 87 | 56.8 (8.5) | 62.07% | 155 (18) | 97 (12) |  |
|  |  |  |  |  | Controls | 97 | 57.2 (9.5) | 49.49% | 133 (10) | 74 (8) |  |
| Li Q et al. | 2006 | Han | hospital | Beijing, Tangshan, Jilin,Shandong | Cases | 345 | 36.3 (6.11) | 53.91% | 157.39 (51.48) | 98.08 (12.82) | 24.18 (1.38) |
|  |  |  |  |  | Controls | 281 | 35.78 (7.36) | 56.58% | 114.4 (11.09) | 73.58 (6.78) | 23.58 (1.91) |
| Hu R et al. | 2006 | Mongolia | population | Urad Back Banner, Inner Mongolia | Cases | 203 | 62.7 (10.6) | 49.6% |  |  | 23.1 (3.1) |
|  |  |  |  |  | Controls | 124 | 59.5 (10.3) | 51.8% |  |  | 24.3 (2.1) |
| Jing J et al. | 2006 | Uygur | population | Turfan city, Xinjiang province | Cases | 354 | 54.49 (10.3) | 35.31% | 163.85 (22.46) | 95.91 (13.15) | 27.92 (4.6) |
|  |  |  |  |  | Controls | 384 | 51.25 (10.22) | 42.19% | 118.99 (12.18) | 74.14 (8.39) | 25.74 (4.18) |
| Chen X et al. | 2007 | Han* | hospital | Wenzhou city, Zhejiang province | Cases | 109 | 58.3 (10.1) | 53.21% |  |  |  |
|  |  |  |  |  | Controls | 378 | 58.1 (8.4) | 54.5% |  |  |  |
| Gai X et al. | 2007 | Han | population | Zhangwu country, Liaoning province | Cases | 136 | 47.56 (10.87) | 30.15% |  |  |  |
|  |  |  |  |  | Controls | 197 | 47.24 (12.08) | 39.09% |  |  |  |
| Chen X et al. | 2008 | Kazakh | population | Barkol county, Xinjiang province | Cases | 241 | 49.54 (12.01) | 45.64% |  |  | 25.04 (3.38) |
|  |  |  |  |  | Controls | 200 | 45.87 (11.22) | 39% |  |  | 24.33 (3.07) |
| Liu W et al. | 2009 | Uygur | population | Turfan city, Xinjiang province | Cases | 269 | 54.56 (10.29) | 38.29% |  |  | 27.68 (4.42) |
|  |  |  |  |  | Controls | 229 | 52.35 (10.93) | 41.92% |  |  | 26.11 (4.34) |

*Abbreviations:* gender (M), gender (males); SBP, systolic blood pressure; DBP, diastolic blood pressure; BMI, body mass index; NA, not available.

Continuous data are expressed as mean (SD) including age, blood pressure and BMI. Gender was expressed as percentage for males.

*The genotype distributions of GNB3 C825T polymorphism was deviated from Hardy-Weinberg equilibrium.

***References***

1. Dai S, Shi J, Ding Q, Wang H, Dong L, et al. (2002) Polymorphism Analysis of 825C/T of the G-protein β3 Subunit in High Risk Population of Hypertension in the Northeast China. ACTA GENETICA SINICA 29:294-298. [Article in Chinese]
2. Wang H, Sun N, Gao Y, Gou S (2003) G protein β3 subunit C825T polymorphism and essential hypertension in Chinese. JOURNAL OF PEKING UNIVERSITY (HEALTH SCIENCES) 35: 423-425. [Article in Chinese]
3. Tan J, Zhu Z, Zhu S, Yu C, Wang L, et al. (2003) The relationship between G-protein β3 subunit gene C825T polymorphism and the pathogenesis of essential hypertension. ACTA ACADEMIAE MEDICINAE MILITARIS TERTIAE 25: 1381-1384. [Article in Chinese]
4. Chen Y, Li G, Li C, Huang X, Ju Z, et al. (2003) Association between G-protein β3 subunit (GNB3) gene C825T polymorphism, hypertension, insulin resistance and obesity. NATIONAL MEDICAL JOURNAL OF CHINA 83: 1229-1232. [Article in Chinese]
5. Wang X, Wang S, Lin R, Jiang X, Cheng Z, et al. (2004) GNB3 gene C825T and ACE gene I/D polymorphisms in essential hypertension in a Kazakh genetic isolate. J Hum Hypertens 18: 663-668.
6. Huang X, Ju Z, Song Y, Zhang H, Sun K, et al. (2003) Lack of association between the G protein beta3 subunit gene and essential hypertension in Chinese: a case-control and a family-based study. J Mol Med 81: 729-735.
7. Huang X, Jiang X, Duan Y, Zhang S, Yao L, et al. (2005) Polymorphism: analysis of 825C/T of the G-protein β3 subunit gene in high risk population of hypertension of Dong nationality group in Huaihua district of Hunan. CHINESE JOURNAL OF BIRTH HEALTH AND HEREDITY 13: 21-22. [Article in Chinese]
8. Zhang J, Li L, Zhang Z, Cui T (2005) Association of G Proteinβ3 Subunit dimorphism with essential hypertension in Chinese. MEDICAL INFORMATION 18: 358-360. [Article in Chinese]
9. Li B, Ge D, Wang Y, Zhao W, Zhou X, et al. (2005) G protein beta 3 subunit gene variants and essential hypertension in the northern Chinese Han population. Ann Hum Genet 69: 468-473.
10. Li D, Hua Q, Pi L (2006) Synergistic Effects between eNOS Gene G894T and GNB3 Gene C825T Polymorphisms in Essential Hypertension. JOURNAL OF CAPITAL UNIVERSITY OF MEDICAL SCIENCES 27: 480-484. [Article in Chinese]
11. Dong H, Li Q, Wang Q, Luo Z (2006) Association analysis between genetic polymorphism of ADD-1 gene and GNB3 gene and essential hypertension. SOTH CHINA JOURNAL OF CARDIOVASCULAR DISEASES 12: 258-261. [Article in Chinese]
12. Li Q, Ci W, Zhang Y, Guo L, Zhu X, et al. (2006) Relationship between essential hypertension and two related gene polymorphism. CHINESE JOURNAL OF PUBLIC HEALTH 22: 1334-1335. [Article in Chinese]
13. Hu R, Zhao S, Niu G, Hu R, Zhang C, et al. (2006) The association research between C825T polymorphism of G proteinums β3 subunit gene and Mongolian patients with essential hypertension. CHINESE JOURNAL OF BIRTH HEALTH & HEREDITY 14:15-17. [Article in Chinese]
14. Jing J, Wang D, Jiao Y, Wang X, Wen H, et al. (2006) Association study on GNB3 gene polymorphism with essential hypertension in Xinjiang Uiygur group. FUDAN UNIVERSITY JOURNAL OF MEDICAL SCIENCES 33: 433-436. [Article in Chinese]
15. Chen X, Wang D, Wu J, Xiong S, Wang C (2007) The relationship between G-protein β3 subunit (GNB3) gene C825T polymorphism and essential hypertension. JOURNAL OF CLINICAL INTERNAL MEDICINE 24: 333-334. [Article in Chinese]
16. Gai X, Shi J, Zhao Y, Dai S, Fu L, et al. (2007) Multivariate analysis on the relationship between G protein β3 subunit gene 825C/T polymorphism and essential hypertension. CHINESE JOURNAL OF EPIDEMIOLOGY 28: 413-414. [Article in Chinese]
17. Chen X, Wang S, Wang X, Lv M, Jin L (2008) Study on the association of predisposing genes with essential hypertension among Kazakhs ethnic group in Xinjiang. CHINESE JOURNAL OF EPIDEMIOLOGY 29: 752-756. [Article in Chinese]
18. Liu W, Yang W, Wang L, Gan Z, Wang X (2009) Association Between ADRB2 Gene, ENaC Gene and GNB3 Gene Polymorphisms and Hypertension in Uygur Population. CHINESE CIRCULATION JOURNAL 24: 446-450. [Article in Chinese]
